# Supplementary material for: Identification and functional analysis of non-coding regulatory small RNA FenSr3 in Bacillus amyloliquefaciens LPB-18
Source: PeerJ. 2023 May 15;11:e15236. doi: 10.7717/peerj.15236 (PMC10194069; doi:10.7717/peerj.15236)
Supplement: Supplemental Information 4 [file peerj-11-15236-s004.zip › KO/CK-vs-T1_map/map04668.html]

KEGG PATHWAY: TNF signaling pathway - Reference pathway


|  |  |
| --- | --- |
| **TNF signaling pathway - Reference pathway** |  |

[
Pathway menu
| Organism menu
| Pathway entry
| Show description
| User data mapping
]

Tumor necrosis factor (TNF), as a critical cytokine, can induce a wide range of intracellular signal pathways including apoptosis and cell survival as well as inflammation and immunity. Activated TNF is assembled to a homotrimer and binds to its receptors (TNFR1, TNFR2) resulting in the trimerization of TNFR1 or TNFR2. TNFR1 is expressed by nearly all cells and is the major receptor for TNF (also called TNF-alpha). In contrast, TNFR2 is expressed in limited cells such as CD4 and CD8 T lymphocytes, endothelial cells, microglia, oligodendrocytes, neuron subtypes, cardiac myocytes, thymocytes and human mesenchymal stem cells. It is the receptor for both TNF and LTA (also called TNF-beta). Upon binding of the ligand, TNFR mediates the association of some adaptor proteins such as TRADD or TRAF2, which in turn initiate recruitment of signal transducers. TNFR1 signaling induces activation of many genes, primarily controlled by two distinct pathways, NF-kappa B pathway and the MAPK cascade, or apoptosis and necroptosis. TNFR2 signaling activates NF-kappa B pathway including PI3K-dependent NF-kappa B pathway and JNK pathway leading to survival.

|  |  |  |
| --- | --- | --- |
| Reference pathway | 184% 150% 122% 100% 82% 67% 55% | 图片下载 |
